# Acute Suppurative and Subacute Thyroiditis: From Diagnosis to Management

**DOI:** 10.3390/jcm14093233

**Published:** 2025-05-07

**Authors:** Tommaso Toschetti, Cecilia Parenti, Ilaria Ricci, Irene Addati, Sonia Diona, Susanna Esposito, Maria Elisabeth Street

**Affiliations:** 1 Department of Medicine and Surgery, University of Parma, 43126 Parma, Italy; tommaso.toschetti@unipr.it (T.T.); cecilia.parenti@unipr.it (C.P.); ilaria.ricci@unipr.it (I.R.); irene.addati@unipr.it (I.A.); sonia.diona@unipr.it (S.D.); susanna.esposito@unipr.it (S.E.); 2 Unit of Paediatrics, University Hospital of Parma, 43126 Parma, Italy

**Keywords:** acute suppurative thyroiditis, subacute thyroiditis, thyroid infection, pyriform sinus fistula, thyroid abscess, dysthyroidism, thyroid gland, thyroid function

## Abstract

**Background**: Acute suppurative thyroiditis (AST) and subacute thyroiditis (SAT) are two distinct inflammatory conditions of the thyroid gland with different clinical presentation and treatment and that recognize different causes. AST is a rare but serious bacterial infection, often associated with congenital anomalies in children, whereas SAT is a self-limiting, post-viral condition that causes temporary thyroid dysfunction. **Methods**: A comprehensive literature review was conducted using PubMed and UpToDate, including systematic reviews, meta-analyses, case series, and case reports. Studies focusing on epidemiology, pathophysiology, clinical presentation, diagnosis, and treatment were selected, with special attention paid to pediatric cases. **Results**: AST accounts for fewer than 1% of thyroid diseases and is more common in children, with pyriform sinus fistulas being present in 21% of cases. It presents with fever, painful neck swelling, and complications such as abscess formation and airway obstruction. Early recognition and prompt management with broad-spectrum antibiotics, ultrasound-guided aspiration, or surgical drainage are crucial. In contrast, SAT can occur at any age but is most common in adult women and typically follows a viral infection. It presents with anterior neck pain and transient thyrotoxicosis and is generally managed with non-steroidal anti-inflammatory drugs or corticosteroids in severe cases. Accurate differential diagnosis is essential to prevent unnecessary interventions. **Conclusions**: Although rare, both AST and SAT require timely diagnosis and tailored treatment strategies to avoid complications. Advances in imaging and the early detection of congenital anomalies have improved AST outcomes, while SAT remains a self-limiting condition that primarily requires symptom management. Further research is needed to better understand risk factors, pathogenesis, and optimal treatment approaches, particularly in pediatric populations and resource-limited settings.

## 1. Introduction

Acute suppurative thyroiditis (AST) and subacute thyroiditis (SAT) represent two different but significant inflammatory diseases of the thyroid gland affecting both children and adults. This review places particular emphasis on the pediatric population while incorporating general data to highlight differences across age groups. Both these conditions present specific diagnostic and therapeutic challenges, especially in pediatric patients, where underlying anatomical abnormalities or systemic predisposition are usually of importance [1]. In fact, the anatomical structure of the thyroid gland, its high iodine content, extensive vascular supply, and fibrous encapsulation offer considerable protection to the gland [2,3]. Differential diagnosis of these two different forms of thyroiditis is recognized to be crucial for management [1,4]. Continued studies concerning the pathogenesis, risk factors, and strategies for the optimum management of both AST and SAT are crucial to improve outcomes in patients and refine approaches to their treatment. Understanding these conditions and addressing gaps in diagnosis and treatment, especially in resource-limited settings, is crucial to reduce the burden and improve outcomes for these conditions.

The current narrative review focuses on the current knowledge available for clinical guidance of both conditions.

## 2. Methods

We conducted a comprehensive literature review using specific search strings in PubMed combining MeSH terms and specific keywords, and in UpToDate. The search aimed to identify all relevant articles discussing acute suppurative thyroiditis (AST) and subacute thyroiditis (SAT). Initially, the search focused on articles published within the last 20 years, with older studies included selectively due to the limited availability of the literature. The search combined MeSH terms and specific keywords such as “pediatric thyroiditis,” “suppurative thyroid abscess,” “pyriform sinus fistula,” and “subacute granulomatous thyroiditis.” Only articles published in English with available full texts were considered. A total of 79 references were ultimately selected based on clinical relevance, methodological clarity, and pertinence to the objectives of the review. These included case reports, case series, clinical reviews, original studies, and expert recommendations (Table 1).

## 3. Epidemiology and Predisposing Conditions

Acute suppurative thyroiditis (AST) and subacute thyroiditis (SAT) are both uncommon conditions with different epidemiological patterns. AST is responsible for less than 1% of all thyroid diseases and is of clinical importance due to its potential for serious complications [5,6,7]. AST is more frequent in children than in adults [3]. Congenital anomalies, as a pyriform sinus fistula or thyroglossal duct remnants, are key anatomical contributors in childhood [5,8,9]. In one case series, it was reported that AST was associated with pyriform sinus fistulae in 21% of the patients analyzed [3]. Among children, AST mostly occurs between the ages of 5 and 15 years [10,11]. In adults, AST is rarer and tends to be associated with immunosuppression secondary to chemotherapy, HIV, long-term steroid use, trauma, and surgical interventions [12]. A review of 200 cases reported that 15% of cases occurred in children but the median age at presentation is 37 years [6]. Although the overall incidence of AST remains stable, improvements in diagnostic imaging techniques have allowed earlier detection of underlying anatomical defects in children, reducing recurrence rates [13]. Similarly, sensitive imaging and laboratory tests have refined the diagnosis of SAT, helping differentiate it from other thyroid disorders. However, delays in diagnosis still occur, especially in low-resource regions, leading to higher complication rates for both conditions [5]. SAT is less frequent in children than in adults but is observed across all ages. It commonly follows viral respiratory infections and shows seasonal variations, with higher rates during viral outbreaks [14]. The gender distribution also differs between AST and SAT. AST has a slight predominance in females, particularly in children, with male-to-female ratios ranging from 1:1.3 to 1:2 likely due to hormonal influences or anatomical differences [5,10]. SAT has a stronger female predominance, particularly in adults, with an M:F ratio reaching 1:4 in some studies [1,15]. This trend could be explained by autoimmune or hormonal factors that predispose women to thyroid inflammation [1,16]. Geographic differences relative to incidence could be explained also by healthcare access and prevalence of risk factors. In developed countries, congenital anomalies are a primary cause of pediatric AST, and advanced imaging helps to attain an early diagnosis [13,17], whereas, in resource-poor regions, AST may arise from less common pathogens such as Mycobacterium tuberculosis or fungal organisms, which reflect environmental and socio-economic factors [18,19]. The occurrence of SAT is influenced by environmental factors that include outbreaks of viral infections including coxsackievirus, mumps, and adenovirus, highlighting a link with preceding systemic illnesses [1].

## 4. Etiology

The occurrence of both AST and SAT depends on the interplay among anatomical susceptibility, microbial organisms, systemic factors, and exogenous influences that challenge the intrinsic resistance of the thyroid gland to infection and inflammation. Trauma or surgery of the neck may contribute to the development of the infection in AST as the integrity of the encapsulation of the gland is breached by such injuries and serves as a portal for the entry of microorganisms into the gland. Some documented causes of AST include foreign body perforation of the esophagus, such as by fish or chicken bones, pointing to mechanical injury as a contributory factor to the infection of the thyroid gland [20,21]. The most common pathogens isolated are *Streptococcus* and *Staphylococcus* species [22]. Of these, *Streptococcus pyogenes* and *Staphylococcus aureus* are the most common, followed by *Staphylococcus epidermis* and *Streptococcus pneumoniae* [3,6,23]. Mixed infections of aerobic and anaerobic bacteria are equally common, especially in cases exhibiting anatomic abnormalities or when abscesses are present [10,24]. In immunocompromised patients, the etiology further extends to include opportunistic pathogens such as *Candida tropicalis* and *Aspergillus* spp., and atypical bacteria such as *Mycobacterium tuberculosis*. These infections often present in an atypical manner and, thus, require adapted therapeutic strategies [25,26,27]. Environmental exposure plays a role in the etiology of AST, particularly in regions where tuberculosis and brucellosis are endemic and can directly or indirectly infect the thyroid gland [26]. The most reported bacterial causes of AST are reported in Table 2.

SAT, as previously mentioned, is generally secondary to an inflammatory process owing to viral infections. Commonly implicated viruses include coxsackievirus, adenovirus, mumps virus, and echovirus, which initiate an immune-mediated response, resulting in thyroid disfunction [1,28]. Cases following SARS-CoV-2 infection have been described in adults only [29]; however, SAT secondary to SARS-CoV-2 infection may occur through direct and/or indirect mechanisms, causing the destruction of thyrocytes [30]. The ability to appreciate that SAT is a post-viral and self-limited condition in nature assists in the avoidance of unwarranted interventional procedures, and treatment can be directed toward symptom alleviation and surveillance for transient thyroid dysfunction, as described in the following paragraphs.

Although rare, there are documented cases where acute suppurative thyroiditis (AST) occurred in the context of underlying thyroid malignancy, particularly papillary thyroid carcinoma (PTC). In such instances, the infectious process may mask an oncological condition. Otani et al. (2018) described a healthy adult woman in whom AST revealed the presence of papillary carcinoma in the affected lobe [31]. Similarly, Kalladi Puthanpurayil et al. (2018) reported a pediatric case where AST was the initial manifestation of PTC [32]. These reports suggest that, especially in atypical or recurrent presentations, oncologic screening should be considered as part of the diagnostic workup, including fine-needle aspiration (FNA) cytology and imaging to rule out coexisting thyroid cancer. Further studies are required to clarify the pathophysiological link between thyroid infections and tumor microenvironment.

### Genetic and Epigenetic Considerations

Evidence suggests that subacute thyroiditis (SAT) may have a genetic predisposition, particularly in individuals with the HLA-B*35 haplotype. This haplotype is found in most SAT cases and may contribute to an exaggerated immune response to viral antigens [33]. Additional susceptibility factors include gene polymorphisms related to immune function and inflammatory pathways, though these associations remain under investigation. Epigenetic mechanisms, such as dysregulated microRNA expression and altered interferon signaling, have also been proposed as factors influencing the severity and recurrence of SAT [15]; however, direct mechanistic evidence is still limited. In contrast, acute suppurative thyroiditis (AST) has not been linked to specific genetic or epigenetic factors.

**Table 2:** Infectious agents that have been identified in acute suppurative thyroiditis [6,24].

Category	Infectious Agents	Comments	References
Bacterial	*Staphylococcus aureus*	The most common bacterial cause: it is often associated with abscess formation.	[34]
	*Streptococcus pyogenes*	Can cause severe cases, especially in children.	[21,35]
	*Streptococcus pneumoniae* *Escherichia coli*	Less common but significant in specific populations. Associated with immunocompromised states or anatomical abnormalities.	[35,36,37]
	*Klebsiella pneumoniae*	Reported in nosocomial infections and immunocompromised patients.	[38]
	*Salmonella* spp.	Rare causes, linked to underlying systemic infections.	
Anaerobic Bacteria	*Fusobacterium* spp.	Reported in cases associated with dental or oropharyngeal infections.	[39]
	*Bacteroides* spp.	They can cause mixed infections with aerobic bacteria.	[40]
Fungal	*Candida albicans*	Occurs in immunocompromised individuals, such as those undergoing chemotherapy.	[19]
Parasitic	*Entamoeba histolytica*	Extremely rare; reported in endemic areas.	[41]
Polymicrobial Infections	Combination of aerobic and anaerobic bacteria	Frequently found in cases with anatomical abnormalities such as pyriform sinus fistula.	[24]
Viral	*Epstein–Barr virus* (EBV)	Rare, usually seen in immunocompromised patients.	[42,43]
	*Cytomegalovirus* (CMV)	Similarly to EBV, in cases with compromised immune systems.	[6]

## 5. Clinical Presentation

AST presents with painful progressive neck swelling, fever, odynophagia, and dysphonia. The physical signs include a tender, hard mass with intact overlying skin, local warmth, and limitation of neck movements. Signs of fluctuation on palpation with erythema may appear in later stages, being suggestive of abscess formation [5,6,44]. Late diagnosis and management of AST may lead to life-threatening complications, which include airway obstruction, internal jugular vein thrombosis, leakage of the abscess into adjacent tissues, and mediastinitis [45,46]. In contrast, SAT often presents with anterior neck pain and tenderness that frequently irradiate to the jaw or ears. As opposed to AST, erythema or fluctuance are usually absent in SAT. The architecture of the gland is disrupted, with the release of preformed thyroid hormones into circulation, often leading to transient thyrotoxicosis during the acute phase of the condition with typical symptoms [4,47]. Overall, it is a self-limiting condition that, over weeks to months, usually resolves on its own without persistent thyroid dysfunction; some patients may exhibit transient hypothyroidism [47]. Untreated SAT, though with minimal risk of resulting acute complications, may, in some very uncommon cases, lead to prolonged thyroid dysfunction or cardiac complications rarely related with prolonged thyrotoxicosis [15]. It can be seen in individuals with subclinical autoimmune thyroiditis or who present recurrent viral infections, although rarely [48].

The different courses indicate that a differential diagnosis is necessary for proper management of both conditions to ensure optimal outcomes. While AST is an emergency that needs urgent intervention to avoid complications, SAT usually has a benign course, and management is mostly symptomatic.

## 6. Diagnosis

The diagnosis of thyroiditis in both AST and SAT relies on a combination of clinical features, laboratory investigations, and imaging. If AST is suspected, laboratory tests should include a complete blood count, inflammatory markers such as C-reactive protein (CRP) and erythrocyte sedimentation rate (ESR), and thyroid function tests. Common findings include leukocytosis, elevated CRP and/or ESR, and increased thyroglobulin levels. Fine-needle aspiration (FNA) is crucial for cytology and bacterial culture to identify the pathogens [22].

Imaging is critical for the diagnosis of AST. Ultrasound is typically the first-line imaging tool, revealing an ill-defined, heterogeneous iso- or hypoechoic mass with peripheral vascularity, more often of the left thyroid lobe. For advanced or unclear cases, cervical computed tomography (CT) scan with contrast or magnetic resonance imaging (MRI) may be needed to determine the extent of the infection and its impact on surrounding tissues [49]. Recurrent AST may warrant barium esophagography or hypopharyngoscopy to detect congenital anomalies sucg as pyriform sinus fistulae [6,50,51].

The diagnosis of SAT relies on clinical, laboratory, and imaging features. Patients commonly present with anterior neck pain, tenderness, and symptoms of transient thyrotoxicosis. Laboratory findings typically show suppressed TSH levels with elevated free T4 and T3 during the thyrotoxic phase, associated with elevated inflammatory markers such as increased ESR and CRP. Unlike AST, in SAT, leukocytosis is not typically present, and cultures are unhelpful [46]. In US imaging, SAT often shows diffusely hypoechoic thyroid tissue with reduced vascularity. Thyroid scintigraphy can help in selected cases, showing reduced iodine uptake during the thyrotoxic phase, and allowing clinicians to differentiate SAT from other hyperthyroid conditions as Graves’ disease [6,49]. Fine-needle aspiration (FNA) cytology is a valuable diagnostic tool in the evaluation of inflammatory thyroid disorders. In acute suppurative thyroiditis (AST), FNA typically reveals a purulent background rich in neutrophilic infiltrates, cellular debris, and sometimes microbial colonies, especially in immunocompromised patients. Necrosis is common, and colloid is often absent. These findings support a diagnosis of bacterial infection and prompt the need for microbiological culture [52]. In contrast, subacute thyroiditis (SAT) shows a granulomatous pattern on cytology. Key findings include multinucleated giant cells, epithelioid histiocytes, disrupted thyroid follicles, and occasional degenerated follicular cells in a background typically poor in colloid. Neutrophils are usually absent or minimal, helping to differentiate SAT from AST and from anaplastic or poorly differentiated thyroid cancers that can also present with pain and inflammation [53]. Histological confirmation, though not always required, reveals non-caseating granulomas and follicular destruction in SAT, while AST shows suppurative necrosis and possible signs of abscess formation. A combined clinical, cytological, and radiological assessment is essential for accurate diagnosis and appropriate management.

## 7. Thyroid Function and Management

As previously described, a careful diagnostic workup is essential to differentiate infectious thyroiditis from subacute thyroiditis to avoid inappropriate management [54]. With regard to thyroid function, it is well-established that the majority of patients with AST present a euthyroid state, which means that their thyroid hormone levels are within the normal range despite the presence of an infection [10]. However, a small proportion of cases may present either hyperthyroidism or hypothyroidism, usually transient and resolving once the underlying infection is treated [12]. The thyrotoxicosis state, though infrequent in the general adult population, is even rarer in pediatric patients, with only a limited number of documented cases reported in the literature [14,54]. The mechanism underlying thyrotoxicosis is ascribed to the release of preformed thyroid hormones from damaged thyroid follicles as a consequence of the infectious process, leading to an excess of circulating thyroid hormones [14]. Clinically, patients may display a range of symptoms commonly associated with thyrotoxicosis, such as palpitations, anorexia, diarrhea, excessive sweating, insomnia, anxiety, tremors, and agitation [54,55]. In these cases, supportive management is essential. Beta-blockers are commonly used to control symptoms such as tachycardia and hypertension, offering symptomatic relief without interfering with thyroid hormone levels [56]. Antithyroid medications, which are indicated in autoimmune hyperthyroidism, are typically unnecessary, as hormone levels usually normalize as the infection resolves [56]. Transient hypothyroidism may also occur in rare cases, often due to extensive follicular destruction that temporarily impairs hormone production. These cases are generally transient, with recovery occurring within approximately three weeks after the infection clears [57]. The specific etiology of AST plays a significant role in determining the risk of thyroid dysfunction. Bacterial SAT in otherwise healthy children generally spares thyroid function. Conversely, rare infections, such as mycobacterial or fungal thyroid infections in immunocompromised children, are more likely to lead to chronic thyroid abnormalities [41,58]. For instance, infections by *Pneumocystis jiroveci* in HIV-infected pediatric patients are associated with hypothyroidism, while some mycobacterial infections initially present with hyperthyroidism before progressing to hypothyroidism [19,41,59]. In subacute thyroiditis, thyroid function is typically characterized by an initial phase of thyrotoxicosis resulting from the release of preformed thyroid hormones due to the inflammation of the gland [28]. This phase can persist for several weeks, extending up to two months, and is followed by a transient euthyroid state and subsequently by a hypothyroid phase which occurs because the gland temporarily loses its ability to uptake iodine and synthesize new hormones. This hypothyroid phase may last for several weeks or months. However, in most cases, normal thyroid function (i.e., euthyroidism) is restored within 6 to 12 months following the onset of the inflammatory condition [28]. In conclusion, the relationship between infectious thyroiditis and thyroid dysfunction is complex and multifactorial. Thyroid function may be transiently altered in response to the infection, with a broad-spectrum of potential outcomes, from euthyroid states to hyperthyroidism, hypothyroidism, or even transient T3 reductions. Identifying the underlying etiology of the infection is crucial for appropriate diagnosis and management. Although uncommon, thyroid storm (TS) represents a life-threatening endocrine emergency that may develop in patients with untreated or severe thyrotoxicosis, including those with subacute thyroiditis (SAT). It is characterized by exaggerated systemic manifestations of thyrotoxicosis, such as hyperpyrexia, tachycardia, severe agitation, confusion, vomiting, and multiorgan dysfunction. Precipitating factors may include systemic infections, trauma, or abrupt discontinuation of antithyroid medications. Although more typical of autoimmune hyperthyroidism, cases of TS following viral or inflammatory thyroiditis have been reported, especially in predisposed individuals [60]. Diagnosis is clinical and based on scoring systems such as the Burch–Wartofsky Point Scale, while laboratory tests confirm severe thyrotoxicosis. Treatment requires aggressive multimodal therapy: beta-blockers (e.g., propranolol) to control adrenergic symptoms, corticosteroids to reduce peripheral conversion of T4 to T3, and supportive care to manage complications. Antithyroid drugs (e.g., propylthiouracil, methimazole) are usually not required in SAT-related TS, since hormone overproduction is not the primary mechanism. Rapid recognition is critical to reduce morbidity and mortality in affected patients [61].

## 8. Antibiotic Therapy for Acute Suppurative Thyroiditis (AST) and Treatment of Subacute Thyroiditis

Without timely intervention, AST can lead to serious complications, including abscess formation, sepsis, and potential long-term thyroid issues. Given the potential for rapid disease progression, broad-spectrum antibiotics are the cornerstone of AST treatment. Therapy usually begins with intravenous (IV) antibiotics to achieve high serum levels quickly [17]. This empirical approach provides broad coverage while waiting for culture results to refine treatment. When the pathogen is still unknown, common choices for empirical antibiotics include amoxicillin-clavulanate, ceftriaxone, and cefazolin. These target common Gram-positive pathogens such as *Streptococcus* and *Staphylococcus* spp., while amoxicillin-clavulanate also provides anaerobic coverage [5]. In cases where anatomical anomalies, such as a pyriform sinus fistula, are suspected, coverage for anaerobic pathogens is critical [62]. Clindamycin and metronidazole are often added to the regimen, either alone or in combination with other antibiotics. Studies report that metronidazole targets mixed infections including anaerobes effectively [11]. Once culture results are available, therapy can be narrowed to the specific pathogen, reducing unnecessary use of broad-spectrum antibiotics and minimizing resistance risks. For instance, vancomycin is indicated for *S. aureus* or methicillin-resistant S. aureus (MRSA) infections, addressing resistant Gram-positive pathogens effectively [12]. If MRSA is suspected or confirmed, linezolid may be considered too. After 5–7 days of IV antibiotics, patients should present clinical improvement (i.e., reduced fever and swelling) and can transition to oral antibiotics [63]. This step reduces hospitalization time and facilitates outpatient care. Segni et al. (2011) documented successful transitions from IV to oral therapy in pediatric AST cases, avoiding surgical intervention and enabling continued recovery at home [64]. A typical antibiotic course for AST lasts 2–3 weeks, depending on the severity of the infection and the presence of complications. Monitoring inflammatory markers, such as CRP and white blood cell count, helps determine the duration of treatment. Clinical improvements and normalized inflammatory markers confirm effective therapy. For recurrent cases or those associated with anatomical anomalies, evaluating and addressing underlying causes, such as pyriform sinus fistulas, is essential to prevent future infections. Table 3 reports the antibiotics and doses used in AST. If an abscess is present, antibiotics alone are often insufficient and require surgical intervention to achieve resolution. Procedures such as ultrasound-guided aspiration or drainage are particularly important in managing these cases effectively. Recurrent AST is often linked to anatomical abnormalities such as pyriform sinus fistulas, so it essential to evaluate children for such defects. Surgical correction of these anomalies can help prevent future episodes [65,66]. A study by She et al. evidenced that combining antibiotics with abscess drainage significantly reduced recurrence rates and shortened hospital stays in children [11]. Initial treatment of SAT often requires non-steroidal anti-inflammatory drugs (NSAIDs), such as ibuprofen, which are effective in reducing inflammation and relieving pain in mild to moderate cases. However, NSAIDs alone may not suffice in the most severe cases. Some authors point out that notwithstanding the use of NSAID, in some cases where symptoms persist, corticosteroids may be required for effective relief [1]. Prednisolone is generally recommended at a dose of 30 mg daily in adults, often achieving remission while minimizing side effects. There is no recommendation for the pediatric population; past case reports typically used 1 to 2 mg/kg/day of prednisone or prednisolone [1,28]. A study by Duan et al. suggested that a short course of one week on corticosteroids, followed by NSAIDs, could be as effective as longer regimens, with fewer side effects and no increased risk of recurrence [67]. Furthermore, they demonstrated that lower doses of prednisolone (20 mg daily, tapered over four weeks) could effectively relieve symptoms, highlighting the possibility of dose adjustment based on patient needs in adults (18 to 70 years old) [67]. Early recognition and management of these conditions are critical to improve outcomes and prevent complications, particularly in children.

## 9. Differential Diagnosis

The clinical similarities among AST, SAT, and other thyroid or cervical conditions require a careful differential diagnosis to prevent inappropriate treatment. A rapidly enlarging thyroid mass with associated pain can mimic AST. However, malignancies are less likely to cause fever or leukocytosis. Fine-needle aspiration can confirm the diagnosis [17]. Infectious mononucleosis is a viral condition that can mimic thyroiditis because of fever, cervical lymphadenopathy, and fatigue. Distinguishing features include splenomegaly and positive heterophile antibody tests. Imaging, particularly ultrasound, typically reveals diffuse lymphadenopathy without thyroid involvement, confirming the diagnosis and ruling out thyroiditis [12]. This differentiation is crucial, as mononucleosis requires supportive management rather than treatment targeted at thyroid inflammation. EBV can, however, be a cause of SAT [74]. Lemierre’s syndrome, characterized by internal jugular vein thrombosis and sepsis, also can present with symptoms such as fever and neck pain, mimicking AST. However, imaging findings show thrombophlebitis rather than a thyroid abscess. This syndrome often originated from oropharyngeal infections, and its management includes prompt antibiotic therapy and sometimes requires anticoagulants [64]. Retropharyngeal abscesses, often seen in children following upper respiratory tract infections, can mimic AST due to symptoms like fever, neck pain, and dysphagia. The distinguishing factor lies in the location of the abscess, which is posterior to the pharynx. Imaging studies, such as lateral neck X-rays or CT scan, reveal widened prevertebral spaces, confirming the diagnosis [18,75]. Unlike AST, the treatment of retropharyngeal abscesses often requires surgical drainage in addition to antibiotics [11,76]. Autoimmune thyroiditis, or Hashimoto thyroiditis, presents with painless goiter and symptoms of hypothyroidism, such as fatigue and cold intolerance, rather than the acute inflammatory signs seen in AST. Laboratory findings, including the absence of inflammatory markers, and the presence of antithyroid antibodies, help confirm this diagnosis. Treatment focuses on thyroid hormone replacement when required [77]. Ectopic thyroid tissue infections, such as thyroglossal duct cyst infections, also present with anterior neck swelling and localized symptoms. Imaging often reveals a midline cystic structure rather than a thyroid gland abscess, differentiating these conditions from AST. Management typically involves surgical excision rather than the antimicrobial therapy required for AST [5,22]. Other head and neck infections, including mastoiditis, cervical lymphadenitis, and salivary gland infections, may also mimic AST due to similar systemic and local symptoms. Imaging studies and fine-needle aspiration cytology are critical for differentiation. For instance, mastoiditis involves the temporal bone, while cervical lymphadenitis and salivary gland infections are localized to lymph nodes or salivary glands [78]. Identifying the precise cause ensures targeted management, such as antibiotics for bacterial infections or surgical intervention when necessary [12,41,79]. Diagnosing AST and SAT requires integrating clinical findings, laboratory results, and imaging.

## 10. Conclusions

**Figure 1:**
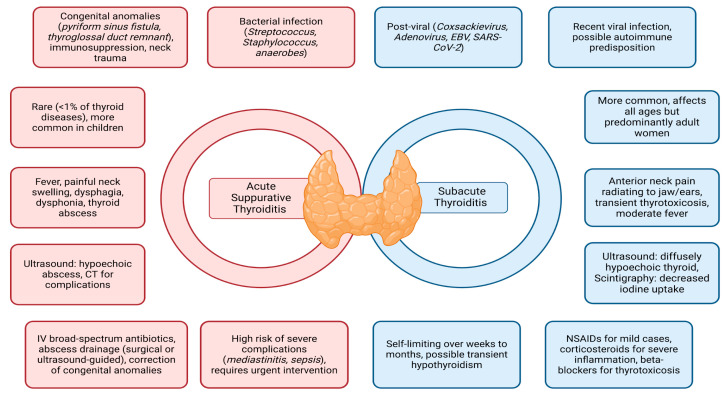
Main features and differences in AST and SAT.

AST and SAT are two distinct inflammatory thyroid conditions, each with unique characteristics in pathophysiology, clinical presentation, and management. While rare, these conditions present significant challenges in diagnosis and treatment, especially in children and individuals with risk factors. Key aspects, including causes, clinical progression, and management strategies, are summarized in Figure 1.

Both conditions underscore the need for a multidisciplinary approach involving endocrinologists, radiologists, surgeons, and infectious disease specialists to ensure comprehensive care and minimize complications. Collaboration across specialties is crucial to enhance care for high-risk populations. Future research should aim to refine diagnostic protocols and treatment strategies, particularly in children and resource-limited settings. Addressing knowledge gaps is essential for improving the prevention, diagnosis, and treatment of these complex thyroid disorders.

## Figures and Tables

**Table 1:** Overview of study types.

Study Types	Articles *n*	Description
Case Reports	34	Description of clinical cases of AST/SAT, with emphasis on pediatric presentations, rare pathogens, complications as abscess or thyrotoxicosis
Case Series/Retrospective Analyses	16	Small- to medium-sized cohorts focusing on clinical features, management, imaging, recurrence
Systematic/Narrative Reviews	11	Comprehensive overviews on pathogenesis, diagnosis, imaging, or therapy (pediatric and general)
Original Research Articles	8	Prospective studies, cytopathologic analyses, drug dosing, imaging, and pharmacokinetics
Guidelines/Clinical Recommendations/Expert Opinions	6	Practical management tools, treatment algorithms, or expert statements
Letters/Editorials/Conference Notes/Book Sections	4	Commentary, update papers, educational or textbook-style summaries

**Table 3:** Dosage guidelines for antibiotics in pediatric acute suppurative thyroiditis (AST).

Antibiotic	Dosage Range (Pediatric)	Administration Frequency	Coverage	References
Amoxicillin-clavulanate	20–40 mg/kg/day of amoxicillin component	Subdivided; every 8 h	Broad-spectrum (Gram-positive, anaerobes)	[68]
Clindamycin	20–40 mg/kg/day	Subdivided; every 6–8 h	Gram-positive, anaerobes	[69]
Ceftriaxone	50–75 mg/kg/day	Once daily	Broad-spectrum (Gram-negative, Gram-positive)	[70]
Cefazolin	50–100 mg/kg/day	Subivided; every 8 h	Gram-positive	[71]
Vancomycin	40 mg/kg/day	Subdivided every 6–8 h	MRSA, Gram-positive	[72]
Metronidazole	15–30 mg/kg/day	Subdivided; every 8 h	Anaerobes	[73]

MRSA: methicillin-resistant *Staphylococcus aureus*.

## Abbreviations

The following abbreviations are used in this manuscript:

AST	Acute suppurative thyroidits
SAT	Subacute thyroiditis
CRP	C-reactive protein
ESR	Erythrocyte sedimentation rate
IV	Intravenous
NSAIDs	non-steroidal anti-inflammatory drugs
MRSA	Methicillin-resistan Staphylococcus aureus
CMV	Cytomegalovirus
EBV	Epstein–Barr Virus
HIV	Human Immunodeficiency Virus
